# Urinary Continence Outcomes after Puboprostatic Ligament Preserving Open Retropubic Radical Prostatectomy at a Sub-Saharan Hospital

**DOI:** 10.1155/2014/986382

**Published:** 2014-12-18

**Authors:** S. Kaggwa, M. Galukande

**Affiliations:** Department of Surgery, School of Medicine, College of Health Sciences, Makerere University, Mulago Hill Road, Kampala, Uganda

## Abstract

*Background*. Open retropubic radical prostatectomy is a commonly performed procedure for clinically localized prostate cancer. The demand for high level functional outcomes after therapy is increasing especially for young age patients; in this regard refinements in the surgical technique have been made. There is limited data to show the success of some of these refinements in resource limited settings. *Methods*. A retrospective clinical study was performed over a 2-year period at Mengo Hospital, Urology Unit. Men with clinically localized prostate cancer and who consented to the procedure were eligible and were recruited. Consequently excluded were those that turned out to have advanced disease and those with severe comorbidities. Patients were followed up for 3 months after surgery. Data was entered using SPSS version 17 and analyzed. *Results*. A total of 24 men with clinically localized prostate cancer underwent open retropubic puboprostatic ligament preserving radical prostatectomy technique. Mean age was 66, range 54–75 years. *Outcome.* Two patients had stress incontinence and three were incontinent at 3 months. The urinary continence recovery rate was 19/24 (79%) at 3 months. *Conclusion*. Preservation of the puboprostatic ligament in open retropubic radical prostatectomy was associated with rapid and a high rate of return to urinary continence among men with clinically localized disease.

## 1. Introduction

Open retropubic radical prostatectomy is a commonly performed procedure for clinically localized prostate cancer [1, 2]. It is increasingly desirable for young age patients, as the demand for high level functional outcomes after therapy becomes more critical [3, 4].

Urinary incontinence and sexual dysfunction represent the most common long term sequelae of radical prostatectomy and the most important determinants of postoperative quality of life [5, 6].

Whereas numerous articles have been published about it, there is dearth of data on the experiences from low resource settings.

We report the functional (urinary continence) outcome of the puboprostatic ligament sparing technique among a group of Ugandan men with clinically localized prostate cancer.

## 2. Methods

A retrospective clinical study was performed over a 2-year period (2012-2013) at Mengo Hospital, Urology Unit. Men with clinically localized prostate cancer defined by a digital rectal examination and transrectal ultrasound and who consented to the procedure were eligible and were operated on and their data were included for analysis. Consequently excluded were those that turned out to have advanced disease and those with severe comorbidities such as cardiac failure or uncontrolled diabetes type 2. The clinically localized prostate cancer was defined in international guidelines [7]. The diagnosis of the disease was established by positive biopsy results.

Patients were followed up for 3 months after surgery in the urology outpatient clinic. Urinary continence was evaluated by a urologist through clinical evaluation; no urodynamic studies were done. Postoperatively the urethral catheters were removed on day 21.

Stress in continence was defined as being “dry” most of the time but a few drops of urine may “leak” while standing or walking to the toilet (to void) incontinence was defined as being “wet” all the time requiring a pad. The follow-up activities included receiving reports from the patients about urinary continence and a direct inquiry concerning ability to control urine plus a midstream urine microscopic examination.

The puboprostatic ligament sparing radical prostatectomy technique used has been described elsewhere [8–10].

*Description of the Technique*. A standard radical retropubic prostatectomy was performed in patients of group A.

The patient was placed in the supine position. A 16 F Foley was inserted, and a supraumbilical midline incision was made. The rectus muscles were separated in the midline, and the transversalis fascia was opened sharply to expose Retzius space. The lymph node dissection was performed at this point if indicated. After the endopelvic fascia had been opened, the division of the puboprostatic ligaments was avoided. A long curved pair of artery forceps was passed in the plane between Santorini plexus and the urethra. It was used to draw two 3/0 vicryl sutures used to ligate the Santorini plexus distally and anchor it on to the pubic symphysis. We adopted the technique described by Kessler et al. to litigate the venous plexus proximally.

The curved Babcock clutch was used to capture the Santorini plexus. Between the Santorini plexus and prostate apex an 8-ligature figure was passed as well as the base of the prostate. The ligated Santorini plexus is sharply transected not the level of the apex but over the lower half of the prostate. Thereafter the plane between the ligated Santorini plexus and the prostate capsule was sharply dissected towards the apex. At the level of the apex, the external rhabdosphincter was progressively transected approximately 2 to 3 millimeters away from the apex. Once the catheter was reached, the urethra was transected on both lateral sides, and the catheter was grasped with a clamp. The catheter was transected as well as the posterior portion of the membranous urethra. Thereafter, the lateral and posterior parts of the prostate were dissected, and, afterwards, the dissection of the vas deferens and removal of the seminal vesicles were performed in both sides. The bladder neck was dissected and reconstructed forming a tennis racket closure by having inverted the bladder mucosa layer from 11 to 1 o'clock. A silicon 18 F Foley catheter was placed, and 6 sutures of the vesicoureteral anastomosis were placed at 1, 3, 5, 7, 9, and 11 o'clock positions and tied without tension. A suction drain was placed, and the incision was closed.

Data were entered using SPSS version 17 and analyzed. Descriptive statistics were derived.

## 3. Results

A total of 24 men with clinically localized prostate cancer underwent open radical retropubic prostatectomy using puboprostatic ligament sparing technique. In Table 1, the descriptive characteristics of the men are outlined including age, functional urinary continence outcomes, and the different variables for pathologic stage (Gleason, PSA, and T stage as well as surgical techniques and approaches).

The urinary continence recovery rate was 19/24 (79%) at 3 months (followup).

One stayed incontinent from the time of catheter removal to 3-month review.

Two deteriorated from being continent to having stress incontinence.

Four improved, two from incontinent to stress incontinence and two from being incontinent to continent.

## 4. Discussion

In this paper we describe our experience of the puboprostatic ligament sparing technique in a series of 24 patients in a urology unit in a low resource setting.

Our data show a rapid return of continence which is similar to what other studies in high income countries described [11–14]. This technique spares the puboprostatic ligaments, which preserves the anterior support of the urethra and therefore contributes to continence [8, 15, 16].

Cancer of the prostate is the commonest malignancy in the male in Uganda [16]. Its incidence rate is 39.2 per 100,000 [17]. It is commonest in the 6th and 7th decade in Africa and elsewhere [17]. This relates well with the mean age of the men in this study.

The primary objective in the management of clinically localized prostate cancer is to cure the disease by total excision or destruction of the cancer while preserving quality of life.

Radical prostatectomy is one of the most common surgical treatment modalities for men with clinically localized prostate cancer [18, 19].

Urinary incontinence and sexual dysfunction are major occurrences that determine the postoperative quality of life [5, 6].

In this study, the urinary inconsistence rate was 11% at 3 months and it would perhaps reduce to 6% by 24 months. The 11% at 3 months is comparable to the rates (5–31%) stated in the literature [20, 21]. These complications are because of the very close anatomical proximity of the prostate gland to the neurovascular bundles and the bladder [8]. Whereas laparoscopic and lately robotic assisted prostatectomy [9] have led to further improvements to the complications following radical prostatectomy, these approaches and techniques for minimally invasive surgery are not readily available in low income countries [22, 23].

In this study like many others we limited ourselves to the immediate postoperative period; we now know that functional outcomes represent a dynamic time dependent process. Men not recovering their urinary continence at a certain point might significantly improve if followed up for longer than 24 months [24]. This effect is termed as conditional survival. 

*Study Limitation*. There was no comparator group, and these data are limited by retrospective design. Despite that limitation, a return to continence after 12 weeks for 79% of individuals was encouraging. Perhaps a longer follow-up period was likely to improve on the urinary continence recovery rate [24].

## 5. Conclusion

This study shows that preservation of puboprostatic ligaments contributes to early recovery rate of urinary continence after open retropubic radical prostatectomy.

## Figures and Tables

**Table 1:** Descriptive characteristics of 24 patients with prostate cancer treated with puboprostatic ligament preservation open retropubic radical prostatectomy between 2011 and 2013.

Characteristics	Number
Age (years)	
Mean	66
Range	54–75
Urinary continence outcome	
Continent	19
Stress	2
Incontinent	3
Pathologic Gleason score	
≤6	5
7	18
≥8	1
Nerve sparing type	
Unilateral	0
Bilateral	24
Pathologic stage	
T_2_	24
Surgery type	
RRP	24
LP	0
PSA (prostate specific antigen, ng/mL)	
Mean	12.7
Median	12.3
Range	5.5–20
Operating time	
Mean	2 hours, 50 minutes
Range	2–4 hours

RRP: retropubic radical prostatectomy.

LP: laparoscopic prostatectomy.
